# Tropical rhodolith beds are a major and belittled reef fish habitat

**DOI:** 10.1038/s41598-020-80574-w

**Published:** 2021-01-12

**Authors:** Rodrigo L. Moura, Maria L. Abieri, Guilherme M. Castro, Lélis A. Carlos-Júnior, Pamela M. Chiroque-Solano, Nicole C. Fernandes, Carolina D. Teixeira, Felipe V. Ribeiro, Paulo S. Salomon, Matheus O. Freitas, Juliana T. Gonçalves, Leonardo M. Neves, Carlos W. Hackradt, Fabiana Felix-Hackradt, Fernanda A. Rolim, Fábio S. Motta, Otto B. F. Gadig, Guilherme H. Pereira-Filho, Alex C. Bastos

**Affiliations:** 1 Instituto de Biologia and SAGE/COPPE, Universidade Federal do Rio de Janeiro, Rio de Janeiro, RJ Brazil; 2 Laboratório de Ecologia Aquática e Educação Ambiental, Universidade Federal Rural do Rio de Janeiro, Três Rios, RJ Brazil; 3 Laboratório de Ecologia e Conservação Marinha, Universidade Federal do Sul da Bahia, Porto Seguro, BA Brazil; 4 Instituto de Biociências, Laboratório de Pesquisa de Elasmobrânquios, Universidade Estadual Paulista, São Vicente, SP Brazil; 5 Laboratório de Ecologia e Conservação Marinha, Instituto Do Mar, Universidade Federal de São Paulo, Santos, SP Brazil; 6 Universidade Federal do Espírito Santo, Vitória, ES Brazil

**Keywords:** Biooceanography, Community ecology, Conservation biology, Ocean sciences

## Abstract

Understanding habitat-level variation in community structure provides an informed basis for natural resources’ management. Reef fishes are a major component of tropical marine biodiversity, but their abundance and distribution are poorly assessed beyond conventional SCUBA diving depths. Based on a baited-video survey of fish assemblages in Southwestern Atlantic’s most biodiverse region we show that species composition responded mainly to the two major hard-bottom megahabitats (reefs and rhodolith beds) and to the amount of light reaching the bottom. Both megahabitats encompassed typical reef fish assemblages but, unexpectedly, richness in rhodolith beds and reefs was equivalent. The dissimilar fish biomass and trophic structure in reefs and rhodolith beds indicates that these systems function based on contrasting energy pathways, such as the much lower herbivory recorded in the latter. Rhodolith beds, the dominant benthic megahabitat in the tropical Southwestern Atlantic shelf, play an underrated role as fish habitats, and it is critical that they are considered in conservation planning.

## Introduction

Coral reefs are among the most biodiverse and productive marine ecosystems, but climate changes and local stressors are driving a rapid and massive global decline^1,2^. Within tropical seascapes, reefs are connected with other benthic megahabitats (i.e. features with dimensions from km to tens of km) through the exchange of propagules and organisms at different life cycle stages, as well as through nutrients and energy fluxes^3^. Connections among reefs, mangroves and seagrass beds enhance biomass and diversity in reef fish assemblages [e.g.,^4^], but the role of other neighboring megahabitats remains unclear, including rhodolith beds, which are among the world’s largest photosynthesizer-dominated benthic communities^5–7^. Due to such major knowledge gaps about the distributions and abundance of organisms in tropical seascapes^8,9^, management and conservation measures are often suboptimal and based on poorly substantiated assumptions about species’ nestedness and turnover patterns^10,11^, among other caveats [e.g.^2,12^]. Comprehensive biodiversity inventories with significant spatial cover may take several decades, but a broader understanding of species-habitat associations provides timely surrogates for management^13^. Shallow water tropical ecosystems can be readily detected by remote sensing and easily sampled with conventional SCUBA in depths of up to 30 m. However, detection and mapping of deeper habitats depends on acoustic surveys and bottom imaging for validation of sonograms, while biological sampling relies largely on mixed-gas diving and submersibles, or fishing^14^. The use of remote underwater video is an emerging approach for sampling fish and benthic assemblages in these less accessible compartments of tropical shelves^9,15^.

Due to extensive commercial exploitation and the relatively large sizes attained by fishes, these vertebrates are among the best-known reef-associated groups^16^. Reef fishes are model organisms to explore evolutionary and biogeographic mechanisms, sustain millions of small-scale fishers, and their global overfishing has bitterly revealed the central role of herbivory, competition and trophic cascades as drivers of coral reef community structure and dynamics^1,17^. As remarked by^18^, reef fishes defy definitions beyond the tautological and are those individuals that live on coral reefs. Globally, approximately 20% of all reef fishes also occur in non-reef habitats^8^, which are used as foraging and spawning grounds, or as recruitment/juvenile habitats. Groupers and snappers, among several other fish families, engage into long-range movements towards nearshore or offshore spawning grounds^19^, and mangroves and seagrass beds can be important habitats for early stages of reef fishes^3,4^. On short time scales, diel movements of diurnal planktivores (e.g. damselfishes) and nocturnal invertivores (e.g. grunts and emperor breams) enhance the coupling among benthic habitats and the water column [e.g.,^3^]. Knowledge gaps about the use of the wider seascape by fish and other organisms, as well as the biological and physical connections between its habitats, still impede conservation planning and management^4,8,20^.

The Eastern South American coast encompasses a relatively isolated, small and species-poor reef Province with high endemism levels and biological communities assembled by species with Caribbean, West African and Indian Ocean affinities^21^. Southwestern Atlantic (SWA) was regarded as a small and fragmented outpost of the “West Indian” reef province until the end of the 20th Century, and a myriad of reef-associated SWA endemics was only described in the past few decades, from fishes to keystone reef-building invertebrates^21^. The size and distribution of SWA reefs are still largely known from nautical charts that only show navigation hazards, but recent multi-level sampling is revealing large and complex mesophotic coralline reefs^6,7^, as well as extensive rhodolith beds [sensu^22^] along more than 30 latitudinal degrees, between 10 and 150 m depths^5^. Indeed, rhodolith beds are the dominant benthic habitat along the tropical SWA continental shelf and one of the world’s largest algae-dominated benthic systems^22^. Nevertheless, the role of tropical rhodolith beds as fish habitats has never been assessed^23^. Due to their broad spatial extension, rhodolith beds may influence ecological processes (e.g. nutrient flow, larval supply) that drive reef fish community structure and dynamics^4^.

Here, we aimed at exploring the importance of rhodolith beds as fish habitats at a range of local to regional scales and depth categories. Our regional-level reef fish survey in the Abrolhos Bank, Brazil, was carried out with stereo Baited Remote Underwater strereo-Videos (BRUVs) across a ~ 30,000 km^2^ mosaic of carbonate hard bottom. The study region comprises SWA’s largest reef complex with (1) fringing reefs around a small archipelago, (2) emerging and quasi-emerging pinnacles distributed in one inshore (10 km off the coast) and one offshore arc (55 km off the coast) totaling ~ 8,800 km^2^, and (3) the world’s largest continuous rhodolith bed (~ 20,900 km^2^)^5,6,24^. The inshore reef arc (~ 10 km offshore) is subjected to high fishing pressure and terrigenous sourced turbidity, while the offshore arc (~ 60 km offshore) is within the Abrolhos National Marine Park (ANMP) and is less exposed to fisheries and land-based stressors. Our standardized survey with BRUVs allowed for richness and biomass estimates for nearshore and mid-shelf reefs, as well as rhodolith beds in depths beyond SCUBA limits. Instead of being marginal (i.e. “suboptimal”) habitats^25,26^, rhodolith beds were found to be major reef fish habitats in the tropical SWA and need to be thoughtfully accounted for conservation planning and marine management.

## Results

We recorded 107 reef fish species (5,155 individuals), 71 (66.4%) in fringing and pinnacles’ reefs and 85 (79.4%) in rhodolith beds (Supplementary Table S1 online). The same richness rank between the two megahabitats was obtained with rarefaction and extrapolation-based estimates (Supplementary Fig. S1 online). Nearly half [49] of all species were habitat generalists that occurred in both megahabitats. Unique occurrences were concentrated in rhodolith beds (36 species, 34%), while reefs had only 22 (21%) unique species (Supplementary Table S1 online). Besides comprising lower species richness, inshore rhodolith beds encompassed few unique species^6^, mainly soft bottom specialists such as flatfishes (*Symphurus* sp. and *Bothus* sp.), sand perches (*Diplectrum* sp.) and the grey triggerfish (*Balistes capriscus*). On the other hand, over half of the species recorded only in rhodolith beds are deeper dwellers, including butterflyfishes (*Chaetodon sedentarius* and *Prognathodes brasiliensis*), planktivorous damselfishes (*Chromis enchrysura* and *C. flavicauda*), flameback angelfish (*Centropyge aurantonotus*), vermillion snapper (*Rhomboplites aurorubens*) and dwarf serranids (*Serranus chionaraia* and *S. phoebe*). Inshore pinnacles had unique species that are biologically connected to estuarine habitats, including mojarras (*Eucinostomus* spp.), mutton snapper (*Lutjanus analis*), crevalle jack (*Caranx hippos*) and Atlantic spadefish (*Chaetodipterus faber*). Unique occurrences in the three offshore pinnacles’ sites comprised the Caribbean reef shark (*Carcharhinus perezi*), sharksucker (*Echeneis naucrates*) and the yellowtail damselfish (*Microspathodon chrysurus*). The fringing reef also had few unique occurrences, including remarkably large schools of chubs (*Kyphosus* spp.).

**Figure 1:**
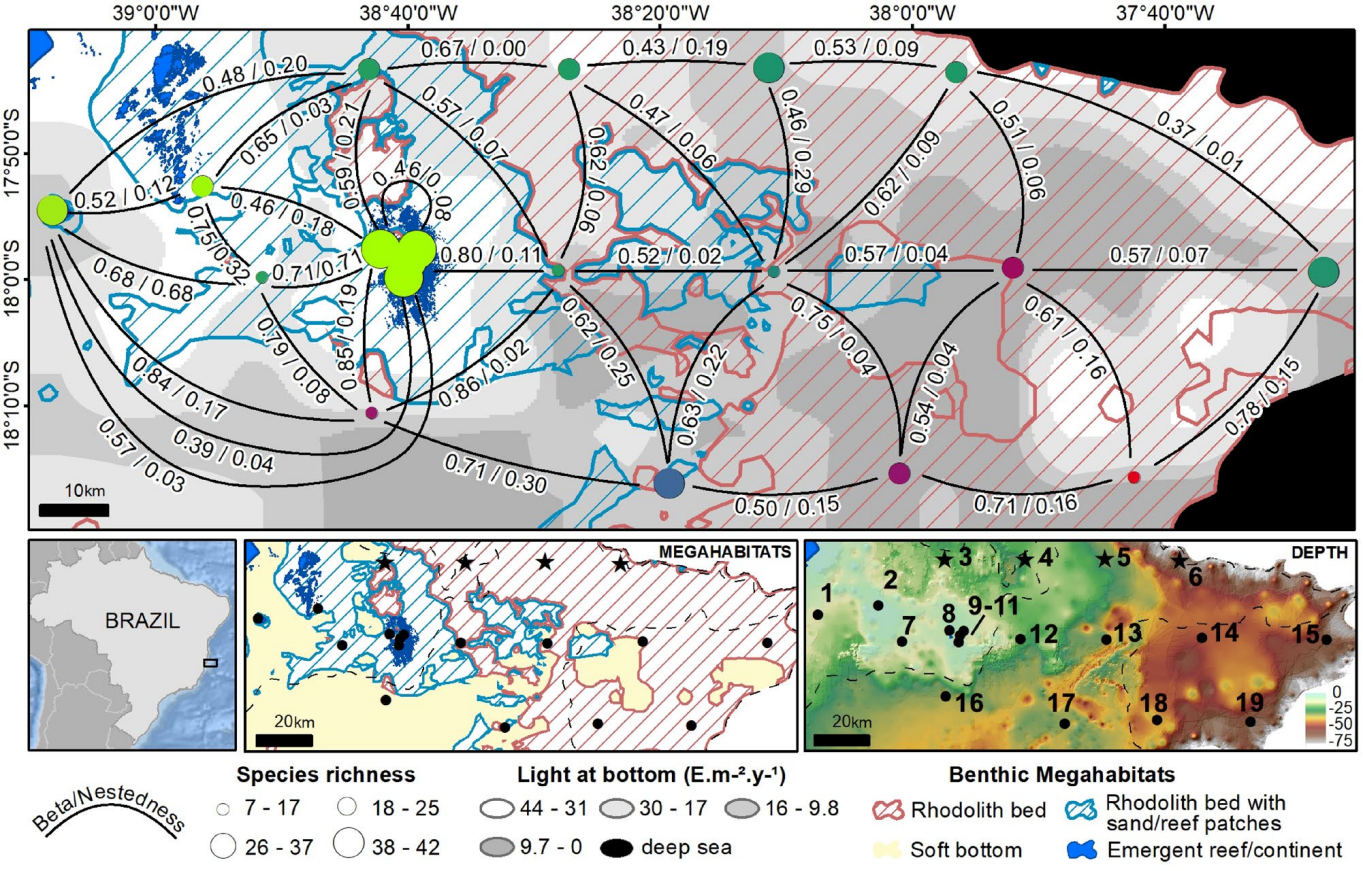
Beta diversity in reef fish assemblages, Abrolhos Shelf. In the larger map (above), color similarity among sites (circles) indicates similar assemblages as measured by their beta diversity distances in a two-dimensional ordination space (see “Methods”) and color circle size corresponds to fish richness; light at bottom and megahabitats were the most important variables in the GDM and are represented as shades of gray scale and stripped patterns, respectively. The arcs connecting sites represent the pairwise total beta/Nestedness components. The lower row shows the localization of the study region, megahabitat distribution and depth (color scale in meters). Sites marked with stars are those depicted in Fig. 5. The number of BRUV deployments at each numbered site (lower right) was: 1 = 16; 2 = 9; 3 = 12; 4 = 7; 5 = 7; 6 = 7; 7 = 4; 8 = 12; 9 = 5; 10 = 9; 11 = 9; 12 = 8; 13 = 7; 14 = 4; 15 = 8; 16- 6; 17 = 6; 18 = 6; 19 = 4 (total = 137 deployments, 1 h each). Map produced with ArcGIS 10.6 (www.esri.com) by J. T. Gonçalves, L. A. Carlos Jr and R. L. Moura.

Considering all sites, the overall compositional variation was relatively high (multi-site beta diversity = 0.88). Most of the variation explained by a generalized dissimilarity model (GDM, overall explained deviance = 46.8%, *p* < 0.000001) was associated with differences in the amount of light reaching the bottom (individual importance = 71.3%, *p* < 0.000001) and megahabitat (pinnacles/fringing vs rhodolith beds; 21.6%, *p* < 0.000001) (Fig. 1; Supplementary Fig. S2 online), while distance among sites was a significant but weaker predictor (1%, *p* < 0.000001). Turnover between pinnacles/fringing reefs and offshore rhodolith sites was relatively high, while inshore rhodolith sites encompassed subgroups of the reef assemblages (Fig. 1). Turnover among reef subtypes, pinnacles’ and fringing reefs, was also small (Fig. 1). The inclusion of percent cover of turf and macroalgae did not significantly improve the explanatory power of the GDM. Turf cover was consistently high in reefs (42.8–55.6%, SD = 6.4%) and positively correlated with light at bottom (r = 0.63), but macroalgae cover in rhodoliths was more variable. Macroalgae were absent from nearshore rhodolith sites and ranged between 2 and 73.7% (SD = 25.4%) in the other sites, with lower values in the deeper and southernmost sites. Variation of reef fish beta diversity across the spatial and environmental gradients is detailed in Supplementary Figure S2 online.

**Figure 2:**
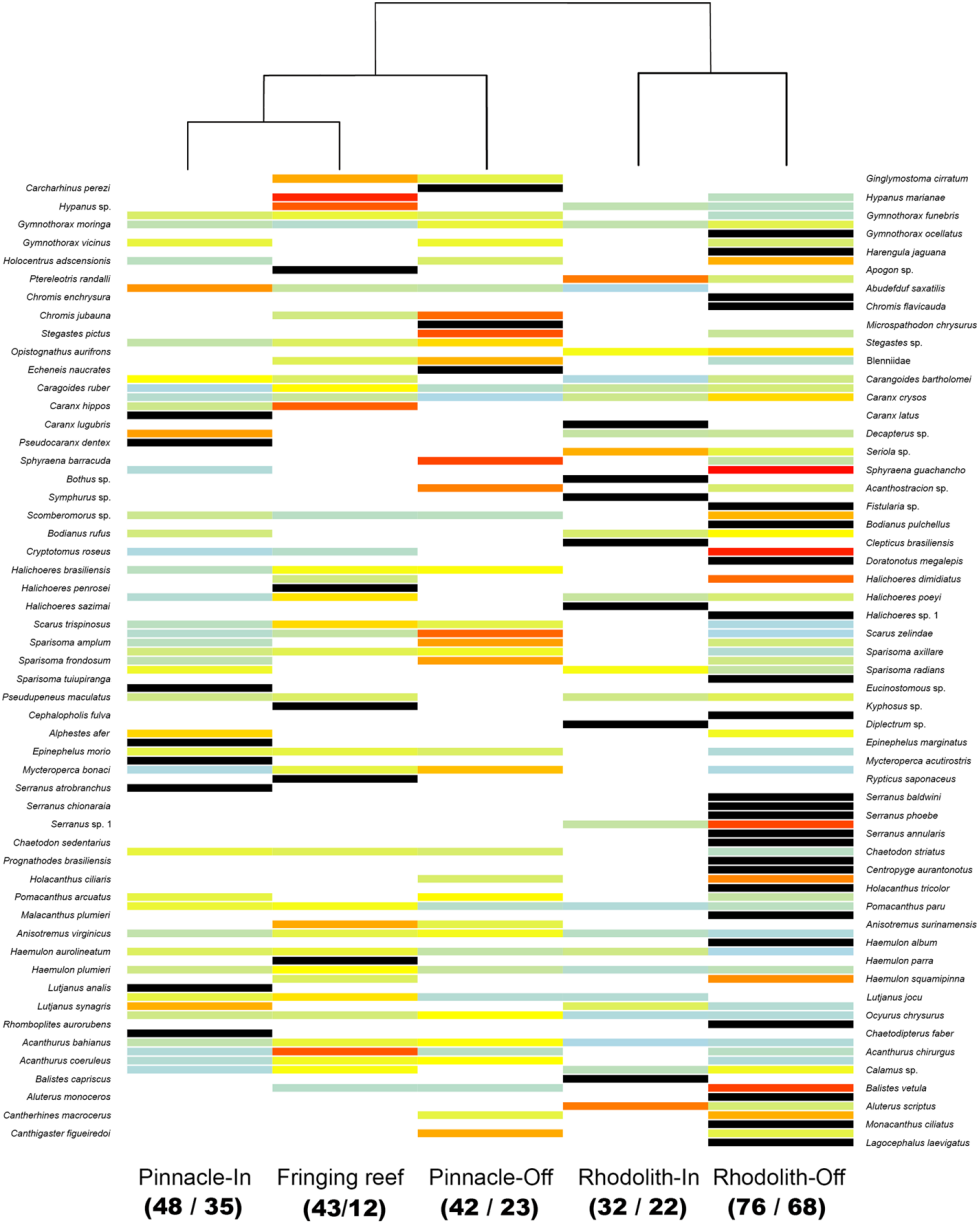
Reef fish abundance in the Abrolhos Shelf. The heatmap was built using relative abundances (rows) weighted by the sampling effort at each sampled strata (columns). Absences are assigned to white, whereas increasing relative abundances are colored from light blue (0.002) to red (0.96). Unique occurrences are shown in black. Column orders were arranged by transforming a matrix of Bray–Curtis dissimilarities among strata into distances (UPGMA). Numbers in parenthesis bellow each habitat column indicate total species richness / number of BRUV deployments. Generated with Freeware R (www.r-project.org), using package gplots.

**Figure 3:**
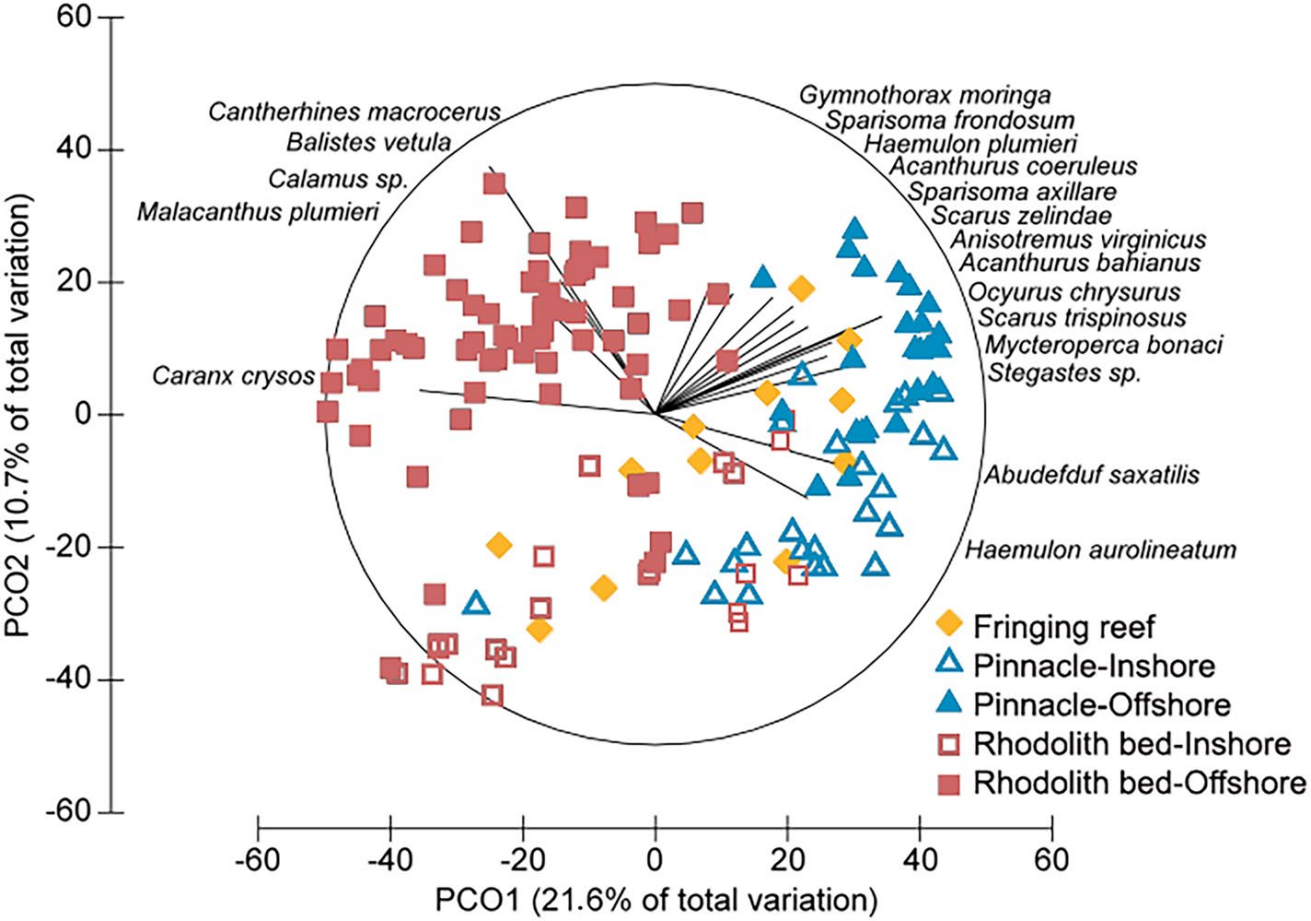
Principal Coordinate Analysis with reef fish biomass data. Sampling strata are color-coded in order to evidence megahabitat and cross-shelf contrasts. Only species with the greatest contribution to the ordination are shown. A PCO with abundance data presented a similar configuration and was omitted. Drawing produced with PRIMER-e v7 (https://www.primer-e.com).

Pinnacles’ and fringing reefs had the highest abundances for 35 fish species that occurred in both benthic megahabitats (71%), while rhodolith beds encompassed the highest abundances for 14 species (29%) that also occurred in reefs (Fig. 2). In terms of fish biomass and abundance, community structure responded to benthic megahabitat and cross-shelf gradients (Fig. 3). The first PCO axis contained 21.6% of the total variation for both biomass and abundance and was largely associated with the two benthic megahabitats (Fig. 3), with negative values associated with rhodolith bed samples. The second PCO axis contained 10.7 and 8.6% of the variation for biomass and abundance, respectively, and was largely associated with the cross-shelf gradient. A smaller assemblage of carnivorous fishes characterized offshore rhodolith samples (Fig. 3), while a richer assemblage with fishes in multiple trophic levels drove reef samples toward positive values in PCO1 (Fig. 3).

**Figure 4:**
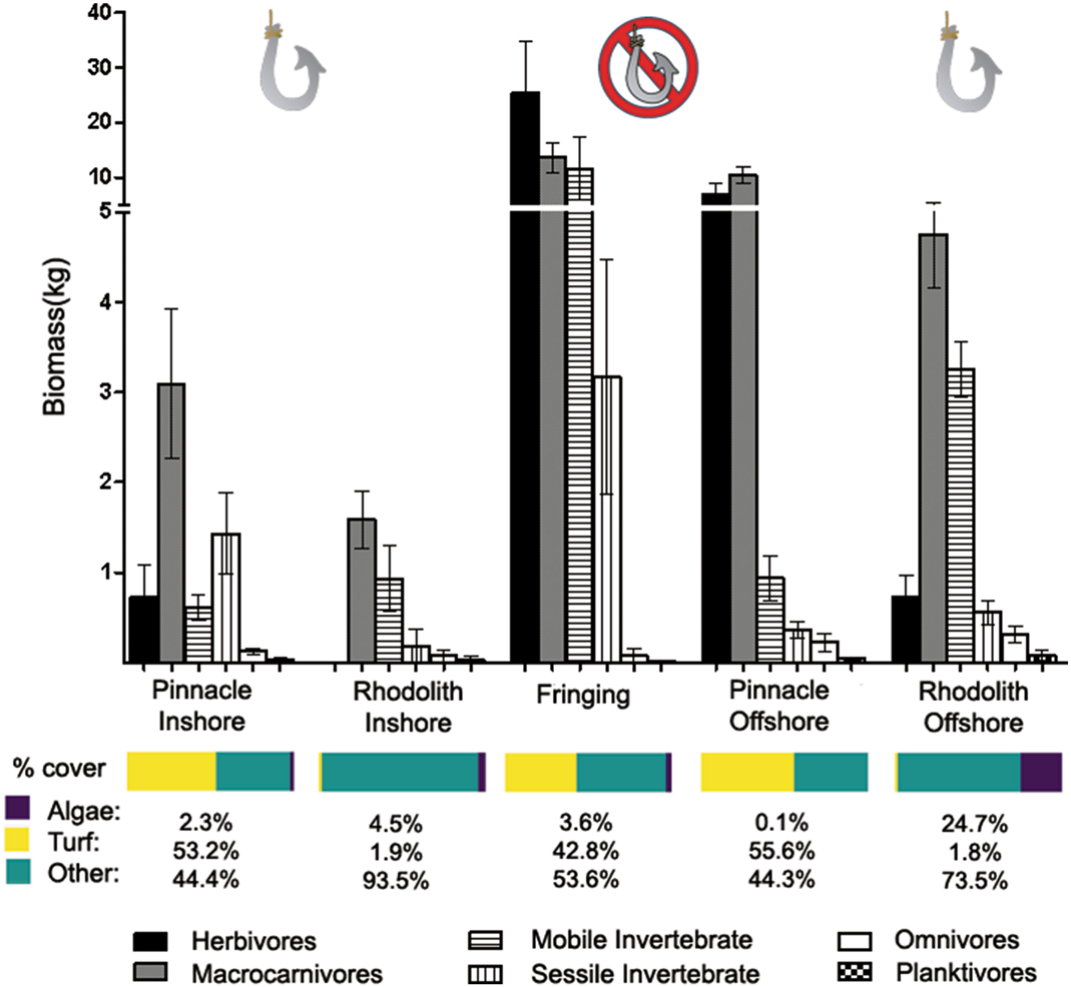
Fish abundance by trophic guild and relative benthic cover in each sampled stratum. Fringing reefs and offshore reef pinnacles are within the no-take enforced zone of the Abrolhos National Marine Park (ANMP). Generated with Freeware R (www.r-project.org).

**Figure 5:**
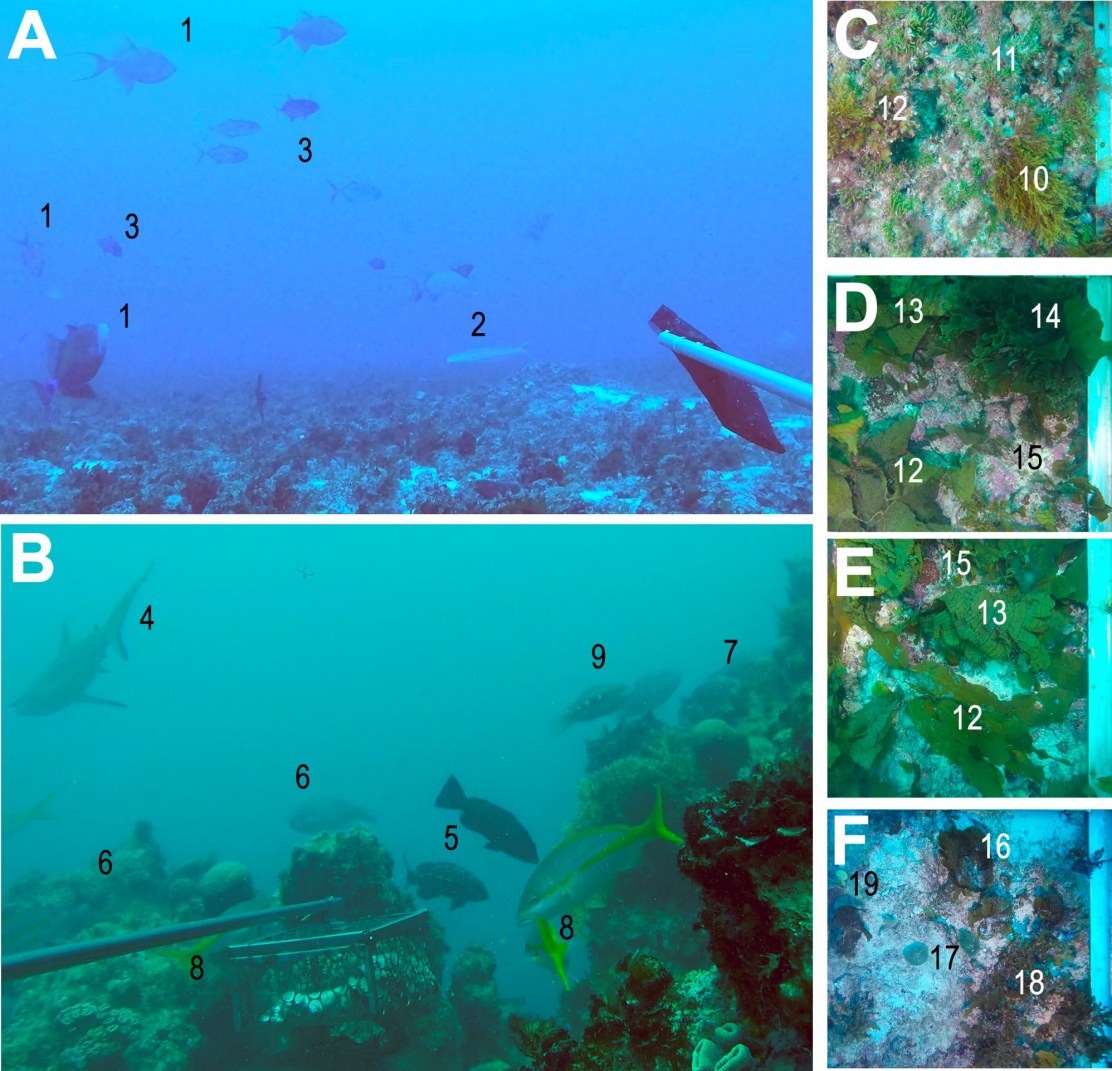
Video frames showing fish and benthic assemblages in reefs (**a**) and rhodolith beds (**b**–**f**). The right row shows orthogonal images from rhodolith bed sites marked with stars in Fig. 1, from the most inshore (**c**) to the most offshore (**f**). Legends: 1 = *Balistes vetula*, 2 = *Malacanthus plumieri*, 3 = *Caranx crysos*, 4 = *Charcharhinus perezi*, 5 = *Mycteroperca bonaci*, 6 = *Scarus trispinosus*, 7 = *Malacanthus plumieri*, 8 = *Ocyurus chrysurus*, 9 = *Scarus zelindae*, 10 = *Dictyota* sp., 11 = *Caulerpa* sp., 12 = *Dictyopteris* sp., 13 = *Stypopodium* sp., 14 = *Zonaria* sp., 15 = Crustose Coralline Algae, 16 = *Lobophora* sp., 17 = *Penicillus* sp., 18 = *Sargassum* sp. Images obtained by R. L. Moura and G. M. Castro.

In comparison to reefs, fish assemblages in rhodolith beds had a distinctive trophic structure, with a remarkable lower abundance of herbivores (Figs. 4, 5). Total biomass in offshore rhodolith sites was consistently higher than that of inshore pinnacles and inshore rhodolith beds (Fig. 4), which were five-fold smaller than in the offshore pinnacles/fringing reefs protected by the ANMP. Despite such sharp biomass contrasts, including the low biomass of herbivores in rhodolith beds, the rank of macrocarnivores, mobile invertebrate feeders, sessile invertebrate feeders and omnivores was similar among reefs and rhodolith beds and cross-shelf strata (Fig. 4). Planktivorous fishes were a minor component of the assemblages. Species endemic to the SWA were numerically important and comprised ~ 18 and 15% of the number of species in rhodolith beds and reefs, respectively. However, SWA-endemics comprised a mere ~ 2% of the fish biomass in rhodolith beds, while they comprised 20% of the fish biomass in reefs. A single SWA-endemic, *Scarus trispinosus*, comprised 17% of the total fish biomass in reefs.

## Discussion

The Abrolhos shelf extends ~ 200 km offshore and is SWA’s most biodiverse region, encompassing a large mid-to-outer shelf hard bottom domain with reefs and rhodolith beds (~ 20,900 km^2^)^5,6^. Fine-sediment dissipative beaches and a large estuary with mangroves dominate the coastline, and terrigenous-mixed sediments predominate in the inner shelf^27^. This large and complex seascape (Fig. 1) comprises a representative experimental setting for understanding the distribution and abundance of reef fishes in different habitats, as well as for exploring the drivers and spatial scaling of beta diversity in reef fish assemblages. The high richness of reef fishes off coral reefs that we found in Abrolhos was unexpected, and sheds new light toward the integration of phenomena that occur at different scales and across distinct habitats and groups of organisms^11,20^. From a practical standpoint, our results are relevant to improve marine management in complex tropical seascapes with rhodolith beds^23^ and other large marginal habitats.

The high richness of reef fishes in rhodolith beds, where fish biomass was smaller than on reefs (Supplementary Fig. S1 online; Fig. 4), seems to be primarily related to the much larger area of rhodolith beds, as well as to the broader depth and cross-shelf range of this hard-bottom habitat, contrasting with reefs. Rather than being a regional idiosyncrasy, the relatively larger area and cross-shelf range of non-reef habitat used by reef fishes seems to be recurrent in tropical shelves across all ocean basins^8,9,23^. However, due to logistical constrains and to the apparent smaller relevance of marginal habitats to fish and other reef-associated organisms, these habitats are still much less sampled than the iconic shallow water reefs^20^, with the exception of mangroves and seagrass beds^3,8,9^.

Compositional variability in biological communities is strongly dependent on spatial scale. Accordingly, beta diversity is expected to be high at biogeographic and local scales, while turnover tends to be lower at regional scales^28,29^. Reef fish assemblages tend to vary sharply at small spatial scales (< 1 km) due to variation in habitat structure, exposure to wave energy and currents, and stochasticity in population dynamics^16^, but may be markedly similar over broader biogeographic areas^21,30^, indicating non-random species associations and convergent ecosystem functioning under similar biotic and abiotic forcing. In Abrolhos, distance among sites was a weak predictor of beta diversity at the seascape level, and turnover between reef sites was also relatively low, akin to the turnover between reefs and inshore rhodolith beds with high amounts of sand and low macroalgae cover (Fig. 1). On the other hand, the relatively high compositional variation at the seascape level responded positively and non-linearly to habitat structure and to the variation in the amount of light reaching the bottom (Supplementary Fig. S2 online). Light at bottom integrates depth and turbidity in a single and ecologically meaningful variable, and captures the major ecological features across coral reef depth gradients^14,31^. The effects of light at bottom over reef fishes seems to be largely indirect and associated with trophic (e.g. grazing and predation on small invertebrates) and non-trophic (e.g. shelter from predators) connections with the benthos^31^.

Reef fishes have a relatively high dispersion potential, both as larvae and as swimming adults, and their distribution at the seascape level is largely constrained by large scale environmental filtering^16,21^. The high turnover recorded at the seascape level, with highly dissimilar reef fish assemblage structures, indicates that reefs and rhodolith beds have distinct functional properties. Turf dominance on reefs (~ 50% of turf cover) may be associated with the high abundance of roving herbivores in this megahabitat, and to the lower turnover between reef sites. Turf algae is the main trophic connection with herbivorous fish, which typically avoid macroalgae^32^, and was positively correlated with light at bottom, which may be associated with the higher reef fish turnovers observed at the seascape level.

Most biological assemblages include a smaller number of abundant species and a long tail of rare species, and coral reef fishes are no exception. Abundance distributions tend to be more skewed toward commonness than the Gaussian and less so than the lognormal distribution, which would be the sum and the product of random variables, respectively^33^. While there were more species with maximum abundances recorded in pinnacles and fringing reefs, nearly one-third of the 49 species recorded in both benthic megahabitats had maximum abundances in rhodolith beds (Fig. 2). This trend is remarkable, as it evidences that reefs may be the marginal (“suboptimal”) habitat for several reef fishes in the SWA. The maximum abundances recorded in rhodolith beds are not related to small juveniles, but the role of rhodolith beds as a critical habitat for juvenile reef fish is unclear and deserves further investigation. We observed small surgeonfish, parrotfish and grunts in rhodolith sites with dense algal canopies (see Fig. 5), which may function as structural refugia against predators^3,4,13^. Akin to mangroves, juvenile reef fish may not depend on rhodolith beds, but the presence of large expanses of hard bottom with dense algal canopies may enhance diversity and biomass in reefs through the exchange of propagules, individuals and nutrients. In addition, rhodolith beds are a better connectivity matrix than soft sediments for adult reef fish migration toward spawning grounds near the shelf edge^19^, as recorded in Abrolhos for the red (*Epinephelus morio*) and black (*Mycteroperca bonaci*) groupers^34^.

The trophic structure of reef fish assemblages was also largely driven by benthic megahabitat and cross-shelf gradients. The low abundance of planktivorous fishes in the Abrolhos reefs has been previously remarked^35^, and is herein confirmed as a regional trend, once this trophic guild was also minor in rhodolith beds. For instance, large schools of forked-tailed plankton-feeding damselfishes (*Chromis* spp.), wrasses (*Clepticus* spp.) and groupers (*Paranthias* spp.), which are common along the entire West Atlantic^16^, are lacking from Abrolhos, where only sparse individuals occur in offshore sites. Several microcarnivores (e.g. *Prognathodes brasiliensis*, *Serranus chionaraia*) and other deeper-water dwellers (e.g. *Rhomboplites aurorubens*, *Chromis enchrysura*) recorded only in offshore rhodolith beds add to the habitat and cross-shelf variation, albeit these species may associate to reef structures across their distribution range^21^. The dissimilar trophic structure of fish assemblages in rhodolith beds was dominated by an impoverished assemblage of benthic-feeding carnivores (e.g. *Calamus* sp., *Cantherhines macrocerus*, *Malacanthus plumieri*) and carnivorous fish that feed both in the bottom and in the water column (e.g. *Balistes vetula* and *Carangoides crysos*, respectively) (Fig. 3). Together with the lower biomass of herbivores (Fig. 4), this trend indicates that most of the energy that flows through fishes in rhodolith beds comes either from small prey captured among the calcareous nodules, or from secondary production in the water column. Relatively longer pelagic food chains in more offshore sites may partially explain the lower reef fish biomass in this megahabitat, added to a clear constrain to fish herbivory within rhodolith beds’ extensive canopies of unpalatable macroalgae such as *Sargassum*, *Lobophora, Padina, Stypopodium* and *Dictyota* (see Fig. 5). Herbivores’ biomass may be underestimated by BRUVs, but the trophic comparisons between habitats (reef vs rhodoliths) should remain valid. However, caution must be taken when comparing baited-video surveys with underwater visual censuses.

Fish biomass in the vast offshore rhodolith beds was consistently higher than that of inshore reefs and rhodolith beds, and seconded that of the outer arc reefs within the ANMP. Considering the several maximum abundances recorded in rhodolith beds (Fig. 2) and the large area of this megahabitat, it is likely that population sizes of several commercially important species (e.g. groupers and snappers) are larger in rhodolith beds than in reefs^9,20^. Fish biomass was five-fold higher in the reefs protected by the ANMP, indicating an overwhelming effect of management regime. The exclusive and frequent occurrence of the endangered Caribbean reef sharks (*Carcharhinus perezi*) within the ANMP adds to the positive effects of the enforced no-take status of this area. Rhodolith beds became the most important reef fishing grounds in the Abrolhos shelf since the 1980s, following the overfishing of inshore reefs^12,31^. However, besides being rarely targeted by enforcement operations, which should regularly restrain illegal fishing gear (e.g. drift nets for lobsters, ‘hookahs’) and seasons’ violations, rhodolith beds and shelf-edge reefs are the least represented habitats in the existing MPA network^6,12^.

It is unlikely that further sampling will change our overall conclusions, nor will have any impact in the recommendations for managers and policy-makers. An additional caveat about the generality of the results presented herein is that rhodoliths occur under a wide spectrum of environmental conditions, from shallow high-energy to deep low-energy settings, from the tropics to polar latitudes^23^, and in ocean basins with very different pools of fish species. Therefore, some rhodolith beds may not be suitable as reef fish habitats. Despite the low SWA-endemic fish biomass (~ 2%) in rhodolith beds, endemics comprised 15% of the fish assemblages in this megahabitat. This finding provides additional support for the idea that rhodolith beds are extremely relevant to the conservation of the biodiversity of the small, unique and highly threatened Brazilian reefs. Rhodolith beds comprise a major but belittled reef fish habitat within the SWA, and should be urgently prioritized in marine spatial planning, licensing and fisheries management. While carbonates’ mining and oil and gas exploitation in reefs are banned in Brazil, these activities are steadily growing in rhodolith beds, which are often categorized as rubble with low importance for biodiversity conservation^36^. Quantitative assessments covering the broad spectrum of reef fish habitats are needed for robust inferences about rarity/conservation status and the habitat range of these vertebrates, which play a major role in healthy ecosystem functioning.

## Materials and methods

### Experimental design

Sampling aiming to assess fish abundance and benthic cover in different megahabitats and cross-shelf strata was carried out in May 2017 and 2018 (summer) in the Abrolhos Shelf (16° 40′–19° 40′ S, 39° 10′–37° 20′ W). Sampling covered 19 sites, six of which in pinnacles’ and fringing reefs, and 13 in rhodolith beds, distributed in a cross-shelf and latitudinal grid (see Fig. 1). We employed six stereo BRUV units similar to those described by^15^, with stainless steel frameworks and two cylindrical PVC camera housings separated by 70 cm and converged at 8°. High-resolution videos were captured with Go Pro Hero 4 cameras (1080i, 60 fps, medium FOV). A flexible PVC arm (1.5 m) was used to hold plastic or metal mesh bags with 800 g of bait (mix of sardines and canned cat food) in front of the camera lens. Replicated 1-h deployments (n = 137) were done between 08:00AM and 16:00PM in order to avoid twilight periods. Sampling effort at each site is detailed in Fig. 1. Each BRUV deployment was separated by at least 450 m, so as to avoid overlaps between bait plumes^15^. Videos were analyzed for 40 min after BRUV systems settled on seafloor. Each system was calibrated before and after each field trip using the software CAL (SeaGIS Pty. Ltd.). Benthic cover was estimated from 5 to 10 standardized (1 × 1 m) orthogonal images of the bottom obtained at each site with a Go Pro camera inside a PVC housing and two light beans (Fig. 5). Benthic images were annotated using 40 random points over each image with the online annotation tool CoralNet (https://coralnet.ucsd.edu/). Abundances and body lengths of each fish species were recorded with the software EventMeasure v3.51 (SeaGIS Pty. Ltd). Relative abundances were based on the MaxN index, a conservative measure that uses the largest number of individuals of each species in a single frame^15^. Since the number of deployments varied among sites, total abundance was divided by the sampling effort at each site. The fork length of all individuals at the MaxN was measured and used to estimate biomasses through conversion constants and length and weight equations provided by FISHBASE (http://www.fishbase.org/, accessed July 2018). Species’ assignments to trophic groups follow^21^.

Light at bottom (E m−^2^ y−^1^) was obtained from the Bio-ORACLE platform (http://www.bio-oracle.org/index.php)^37^, comprising post-processed data by GlobColour (http://globcolour.info) merged with MERIS/MODIS/SeaWiFS data, with a spatial resolution of 9.2 km. Geographical distances among sites and offshore distance were calculated from the geographical coordinates of the sampling sites using the ArcGis platform (ESRI). Bathymetry and megahabitat distribution were obtained from^6,24^. Distribution of emerging and quasi-emerging reefs was obtained from the interpretation and semiautomatic classification of an orbital image (IKONOS satellite, 4 m spatial resolution, 4 spectral bands including NIR, radiometric resolution of 16 bits) in a digital image processing environment. The diffuse attenuation coefficient of light at 490 nm (Kd490) was used as a proxy for turbidity and was obtained from NOAA’s Easier Access to Scientific Data database (https://coastwatch.pfeg.noaa.gov/erddap), at 4 km spatial resolution.

### Statistical analysis

Species richness estimates integrated rarefaction and extrapolation curves using the abundance-based estimator in the inext package^38^. Samples were standardized based on total abundance and extrapolated to 2,000 individuals in order to facilitate comparisons between megahabitats (Supplementary Fig. S1 online). A Principal Coordinates Analysis (PCO) based on a Bray–Curtis dissimilarity matrix was used to explore the spatial variation in assemblage structure, using log-transformed (x + 1) abundance and biomass data. A Spearman’s rank cutoff value of 0.34 was chosen for both data sets in order to show only species with a greater contribution to the ordination.

Total beta diversity was calculated with the Sørensen dissimilarity index and decomposed into the Simpson index, which is related to spatial turnover of species, and the nestedness component^26^. This latter is defined as variation in composition due to species loss/gain, causing composition at species‐poor sites to be subsets of richer sites^37^. Calculations were conducted using the package betapart^39^. Both turnover and nestedness were calculated between all site pairs (see Fig. 1). Beta diversity values were regressed against the spatial (distance offshore, geographic distance among sites and megahabitat types) and environmental (intensity of light at the bottom, turbidity (Kd490), turf percentage cover, macroalgae cover and depth) gradients using generalized dissimilarity modelling (GDM)^40^, implemented by the gdm package (version 1.4.2;^41^). The importance of each variable, as described in the results, was measured as the percent change in the explained deviance observed in the full model (i.e. with all significant variables) and the deviance explained by a model fit only with the individual variable. The output of the model containing only the significant explanatory gradients (megahabitat and light at bottom) was used to predict community dissimilarity for each sampling site. The scores from a multidimensional scaling (MDS) between the predicted beta diversity values among sampling locations were used to assign colors to those sites based on their position in this two-dimensional ordinal space^40^. The same colors for each site were used in Fig. 1, represented against the main environmental drivers of beta diversity across the study region, namely light at bottom and megahabitat type.

## Data availability

Data and scripts are provided as supplemental materials.

## Supplementary Information

Supplementary Information.
