# Supplementary material for: Tropical rhodolith beds are a major and belittled reef fish habitat
Source: Sci Rep. 2021 Jan 12;11:794. doi: 10.1038/s41598-020-80574-w (PMC7804296; doi:10.1038/s41598-020-80574-w)
Supplement: Supplementary file 1 — Supplementary Information. [file 41598_2020_80574_MOESM1_ESM.docx]

**Supplementary Information**

**Tropical rhodolith beds are a major and belittled reef fish habitat**

Rodrigo L. Moura^1,*,†^, Maria L. Abieri^1,†^, Guilherme M. Castro^1^, Lelis A. Carlos-Júnior^1^, Pamela M. Chiroque-Solano^1^, Nicole C. Fernandes^1^, Carolina D. Teixeira^1^, Felipe V. Ribeiro^1^, Paulo S. Salomon^1^, Matheus O. Freitas^1^, Juliana T. Gonçalves^1^, Leonardo M. Neves^2^, Carlos W. Hackradt^3^, Fabiana Felix-Hackradt^3^, Fernanda A. Rolim^4^, Fábio S. Motta^5^, Otto B. F. Gadig^4^, Guilherme H, Pereira-Filho^5^, Alex C. Bastos^6^

^1^ Instituto de Biologia and SAGE/COPPE, Universidade Federal do Rio de Janeiro, Rio de Janeiro, RJ, Brazil

^2^ Laboratório de Ecologia Aquática e Educação Ambiental, Universidade Federal Rural do Rio de Janeiro, Três Rios, RJ, Brazil

^3^ Laboratório de Ecologia e Conservação Marinha, Universidade Federal do Sul da Bahia, Porto Seguro, BA, Brazil

^4^ Instituto de Biociências, Laboratório de Pesquisa de Elasmobrânquios, Universidade Estadual Paulista, São Vicente, SP, Brazil

^5^ Laboratório de Ecologia e Conservação Marinha, Instituto do Mar, Universidade Federal de São Paulo, Santos, SP, Brazil

^6^ Universidade Federal do Espírito Santo, Vitória, ES, Brazil

* [moura.uesc@gmail.com](mailto:moura.uesc@gmail.com)

† These authors contributed equally to this work


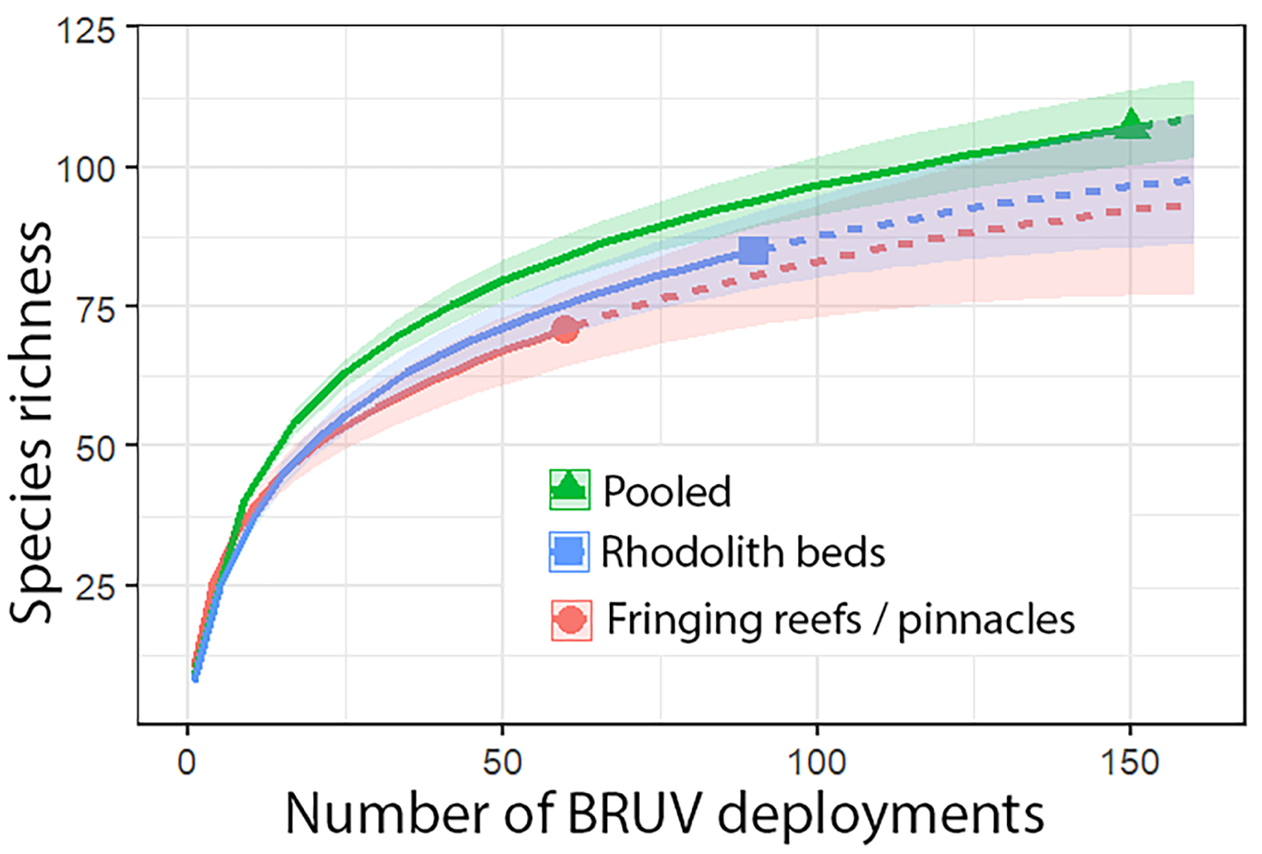


Supplementary Figure S1. **Rarefaction and extrapolation curves (abundance-based) showing the relatively higher reef fish richness in rhodolith beds**.


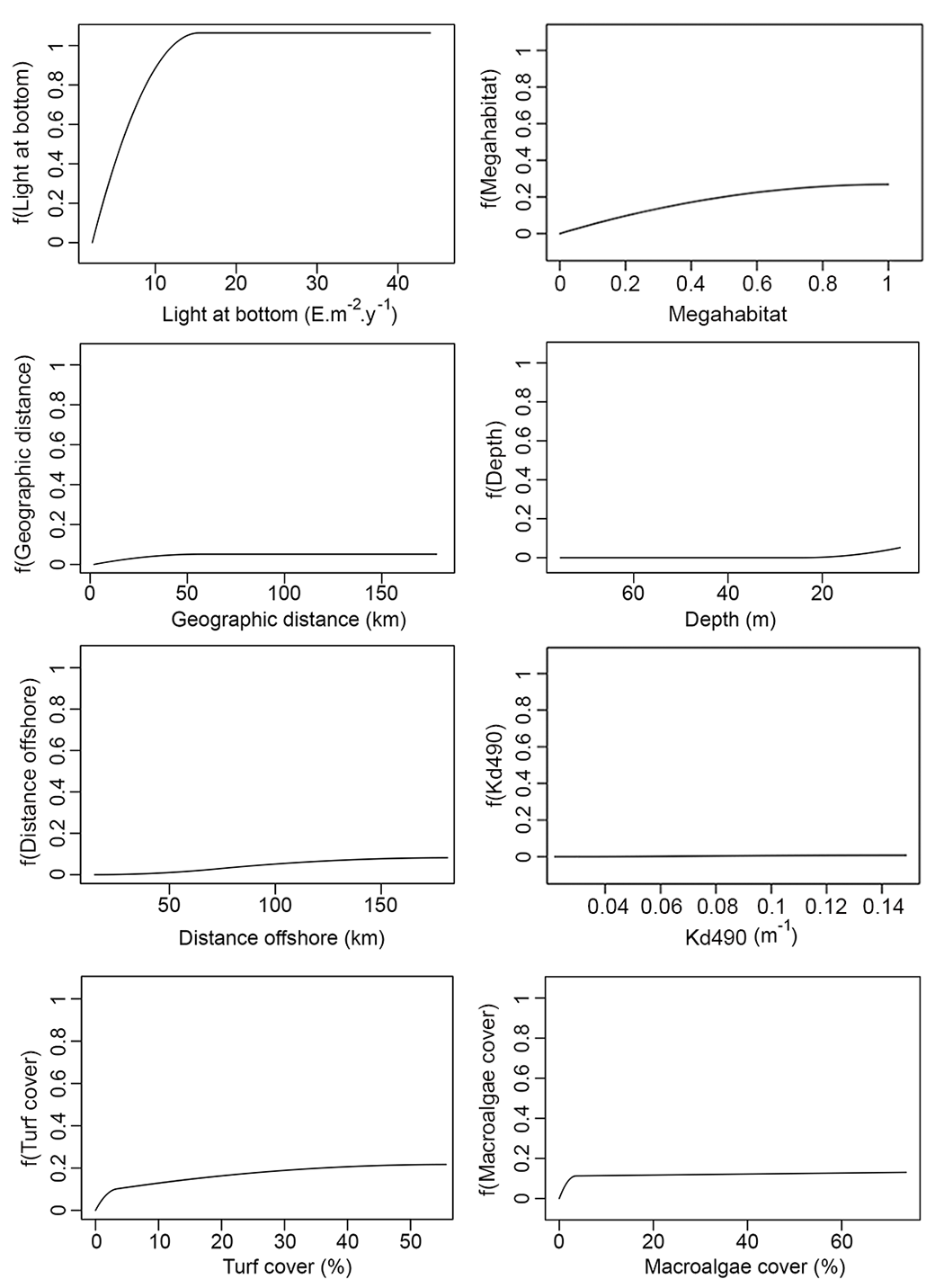


Supplementary Figure S2. **Partial responses of beta diversity (Sørensen dissimilarity index) measured as I-spline functions of the individual spatial/environmental gradients used in the generalized dissimilarity model (GDM) with reef fish data from the Abrolhos Shelf**. The maximum height of each plot indicates the amount of total beta diversity associated with each gradient, holding all other variables constant, and the slope indicates how the beta diversity rates vary along each gradient (see reference *3*)

| Supplementary Table S1. Reef fish traits and abundance patterns in the Abrolhos Shelf, Brazil (database) | | | | | | | | |
| --- | --- | --- | --- | --- | --- | --- | --- | --- |
| **Family** | **Species** | **Trophic guild** | **Max depth (m)** | **SWA endemic** | **Relative abundance ± SE** | | **Biomass (kg) ± SE** | |
|  |  |  |  |  | **Larger reef structures** | **Rhodolith beds** | **Larger reef structures** | **Rhodolith beds** |
| Ginglymostomatidae | *Ginglymostoma cirratum* | MCAR | 130 | No | 0.03 ± 0.02 | 0 | 0.02 ± 0.02 | 0 |
|  |  |  |  |  |  |  |  |  |
| Carcharhinidae | *Carcharhinus perezi* | MCAR | 65 | No | 0.23 ± 0.07 | 0 | 1.17 ± 0.37 | 0 |
|  |  |  |  |  |  |  |  |  |
| Dasyatidae | *Hypanus marianae* | MINV | 15 | Yes | 0.03 ± 0.02 | 0.01 ± 0.01 | 0.06 ± 0.04 | 0.02 ± 0.02 |
|  | *Hypanus* sp*. aff. americanus* | MINV | 65 |  | 0.07 ± 0.03 | 0.03 ± 0.02 | 1.85 ± 1.23 | 0.1 ± 0.07 |
|  |  |  |  |  |  |  |  |  |
| Muraenidae | *Gymnothorax funebris* | MCAR | 70 | No | 0.13 ± 0.04 | 0.02 ± 0.02 | 0.81 ± 0.48 | 0.01 ± 0.01 |
|  | *Gymnothorax moringa* | MCAR | 200 | No | 0.28 ± 0.06 | 0.39 ± 0.07 | 0.48 ± 0.17 | 0.22 ± 0.04 |
|  | *Gymnothorax ocellatus* | MCAR | 160 | No | 0 | 0.03 ± 0.02 | 0 | 0.01 ± 0.01 |
|  | *Gymnothorax vicinus* | MCAR | 186 | No | 0.07 ± 0.03 | 0.04 ± 0.02 | 0.11 ± 0.06 | 0.03 ± 0.01 |
|  |  |  |  |  |  |  |  |  |
| Clupeidae | *Harengula jaguana* | PLANK | 22 | No | 0 | 0.39 ± 0.39 | 0 | 0.04 ± 0.04 |
|  |  |  |  |  |  |  |  |  |
| Holocentridae | *Holocentrus adscensionis* | MINV | 200 | No | 0.07 ± 0.04 | 0.24 ± 0.08 | 0.01 ± 0.01 | 0.04 ± 0.02 |
|  |  |  |  |  |  |  |  |  |
| Apogonidae | *Apogon* sp. | PLANK | 100 | No | 0.02 ± 0.02 | 0 | 0.01 ± 0.01 | 0 |
|  |  |  |  |  |  |  |  |  |
| Microdesmidae | *Ptereleotris randalli* | PLANK | 60 | Yes | 0 | 0.04 ± 0.03 | 0 | 0.01 ± 0.01 |
|  |  |  |  |  |  |  |  |  |
| Pomacentridae | *Abudefduf saxatilis* | OMNI | 20 | No | 2.95 ± 0.73 | 0.01 ± 0.01 | 0.1 ± 0.02 | 0.01 ± 0.01 |
|  | *Chromis enchrysura* | PLANK | 124.5 | No | 0 | 0.52 ± 0.29 | 0 | 0.01 ± 0 |
|  | *Chromis flavicauda* | PLANK | 120 | Yes | 0 | 0.01 ± 0.01 | 0 | 0 |
|  | *Chromis jubauna* | PLANK | 71 | Yes | 0.13 ± 0.09 | 0 | 0.01 ± 0.01 | 0 |
|  | *Microspathodon chrysurus* | HERB | 120 | No | 0.02 ± 0.02 | 0 | 0.01 ± 0.01 | 0 |
|  | *Stegastes pictus* | HERB | 85 | Yes | 0.62 ± 0.26 | 0.24 ± 0.08 | 0.01 ± 0.01 | 0.01 ± 0 |
|  | *Stegastes* sp. | HERB | 55 | No | 1.15 ± 0.21 | 0 | 0.05 ± 0.01 | 0 |
|  |  |  |  |  |  |  |  |  |
| Opisthognathidae | *Opistognathus* sp.2 aff *aurifrons* | PLANK | 65 | Yes | 0 | 0.06 ± 0.04 | 0 | 0.01 ± 0.01 |
|  |  |  |  |  |  |  |  |  |
| Blenniidae |  | OMNI |  | No | 0.08 ± 0.04 | 0.01 ± 0.01 | 0.01 ± 0.01 | 0.01 ± 0.01 |
|  |  |  |  |  |  |  |  |  |

| Echeneidae | *Echeneis naucrates* | MCAR | 50 | No | 0.02 ± 0.02 | 0 | 0.01 ± 0.01 | 0 |
| --- | --- | --- | --- | --- | --- | --- | --- | --- |
|  |  |  |  |  |  |  |  |  |
| Carangidae | *Carangoides bartholomaei* | MCAR | 70 | No | 0.63 ± 0.26 | 0.38 ± 0.14 | 0.15 ± 0.06 | 0.23 ± 0.09 |
|  | *Carangoides ruber* | MCAR | 106 | No | 0.28 ± 0.11 | 0.48 ± 0.16 | 0.12 ± 0.05 | 0.29 ± 0.08 |
|  | *Caranx crysos* | MCAR | 100 | No | 0.50 ± 0.18 | 4.11 ± 0.82 | 0.18 ± 0.06 | 1.38 ± 0.24 |
|  | *Caranx hippos* | MCAR | 350 | No | 0.05 ± 0.03 | 0 | 0.35 ± 0.24 | 0 |
|  | *Caranx latus* | MCAR | 140 | No | 0.02 ± 0.02 | 0 | 0.01 ± 0.01 | 0 |
|  | *Caranx lugubris* | MCAR | 350 | No | 0 | 0.01 ± 0.01 | 0 | 0.03 ± 0.03 |
|  | *Decapterus* sp. | MCAR | 90 | No | 1.05 ± 1.05 | 0.61 ± 0.46 | 0.02 ± 0.02 | 0.11 ± 0.1 |
|  | *Pseudocaranx dentex* | PLANK | 230 | No | 0.05 ± 0.05 | 0 | 0.01 ± 0.01 | 0 |
|  | *Seriola* sp. | MCAR | 160 | No | 0 | 0.09 ± 0.05 | 0 | 0.23 ± 0.12 |
|  |  |  |  |  |  |  |  |  |
| Sphyraenidae | *Sphyraena barracuda* | MCAR | 100 | No | 0.03 ± 0.02 | 0.01 ± 0.01 | 0.17 ± 0.14 | 0.08 ± 0.08 |
|  | *Sphyraena guachancho* | MCAR | 100 | No | 0.02 ± 0.02 | 0.72 ± 0.72 | 0.01 ± 0.01 | 0.16 ± 0.16 |
|  |  |  |  |  |  |  |  |  |
| Bothidae | *Bothus* sp. | MINV | 121 | No | 0 | 0.03 ± 0.02 | 0 | 0.01 ± 0.01 |
|  |  |  |  |  |  |  |  |  |
| Ostraciidae | *Acanthostracion* sp. | OMNI | 80 | No | 0.02 ± 0.02 | 0.01 ± 0.01 | 0.01 ± 0.01 | 0.01 ± 0.01 |
|  |  |  |  |  |  |  |  |  |
| Cynoglossidae | *Symphurus* sp. | MINV | 97 | No | 0 | 0.01 ± 0.01 | 0 | 0.01 ± 0.01 |
|  |  |  |  |  |  |  |  |  |
| Fistulariidae | *Fistularia* sp. | MCAR | 200 | No | 0 | 0.06 ± 0.02 | 0 | 0.01 ± 0.01 |
|  |  |  |  |  |  |  |  |  |
| Scombridae | *Scomberomorus* sp. | MCAR | 140 | No | 0.13 ± 0.07 | 0.51 ± 0.50 | 0.14 ± 0.08 | 0.11 ± 0.08 |
|  |  |  |  |  |  |  |  |  |
| Labridae | *Bodianus pulchellus* | MINV | 120 | No | 0 | 0.02 ± 0.02 | 0 | 0.01 ± 0.01 |
|  | *Bodianus rufus* | MINV | 70 | No | 0.02 ± 0.02 | 0.08 ± 0.04 | 0.01 ± 0.01 | 0.04 ± 0.02 |
|  | *Halichoeres brasiliensis* | MINV | 60 | Yes | 0.28 ± 0.11 | 0 | 0.02 ± 0.01 | 0 |
|  | *Halichoeres dimidiatus* | MINV | 71 | Yes | 0.02 ± 0.02 | 0.23 ± 0.07 | 0.01 ± 0.01 | 0.01 ± 0.01 |
|  | *Halichoeres penrosei* | MINV | 66 | Yes | 0.03 ± 0.03 | 0 | 0.01 ± 0.01 | 0 |
|  | *Halichoeres poeyi* | MINV | 71 | No | 0.68 ± 0.31 | 1.20 ± 0.26 | 0.01 ± 0 | 0.02 ± 0 |
|  | *Halichoeres sazimai* | MINV | 190 | Yes | 0 | 0.02 ± 0.02 | 0 | 0.01 ± 0.01 |
|  | *Halichoeres* sp.1 | MINV | 71 | No | 0 | 0.01 ± 0.01 | 0 | 0.01 ± 0.01 |
|  | *Clepticus brasiliensis* | PLANK | 62 | Yes | 0 | 0.04 ± 0.04 | 0 | 0.01 ± 0.01 |
|  | *Cryptotomus roseus* | HERB | 66 | No | 0.05 ± 0.04 | 1.88 ± 0.43 | 0.01 ± 0.01 | 0.02 ± 0 |
|  | *Doratonotus megalepis* | MINV | 16 | No | 0 | 0.01 ± 0.01 | 0 | 0.01 ± 0.01 |
|  | *Scarus trispinosus* | HERB | 30 | Yes | 3.13 ± 0.96 | 0.09 ± 0.08 | 5.43 ± 1.97 | 0.06 ± 0.05 |
|  | *Scarus zelindae* | HERB | 55 | Yes | 0.80 ± 0.19 | 0.01 ± 0.01 | 0.27 ± 0.09 | 0.01 ± 0.01 |
|  | *Sparisoma amplum* | HERB | 57 | Yes | 0.13 ± 0.05 | 0.08 ± 0.06 | 0.1 ± 0.04 | 0.15 ± 0.13 |
|  | *Sparisoma axillare* | HERB | 45 | Yes | 0.83 ± 0.23 | 0.09 ± 0.06 | 0.22 ± 0.12 | 0.06 ± 0.04 |
|  | *Sparisoma frondosum* | HERB | 45 | Yes | 0.33 ± 0.13 | 0.17 ± 0.06 | 0.17 ± 0.07 | 0.14 ± 0.05 |
|  | *Sparisoma radians* | HERB | 12 | No | 0.02 ± 0.02 | 0.02 ± 0.02 | 0.01 ± 0.01 | 0.01 ± 0.01 |
|  | *Sparisoma tuiupiranga* | HERB | 71 | Yes | 0 | 0.37 ± 0.16 | 0 | 0.02 ± 0.01 |
|  |  |  |  |  |  |  |  |  |
| Gerreidae | *Eucinostomus* sp*.* | MINV | 25 | No | 0.02 ± 0.02 | 0 | 0.01 ± 0.01 | 0 |
|  |  |  |  |  |  |  |  |  |
| Mullidae | *Pseudupeneus maculatus* | MINV | 90 | No | 0.33 ± 0.14 | 0.67 ± 0.19 | 0.02 ± 0.01 | 0.09 ± 0.03 |
|  |  |  |  |  |  |  |  |  |
| Kyphosidae | *Kyphosus* sp. | HERB | 55 | No | 0.43 ± 0.40 | 0 | 1.23 ± 1.11 | 0 |
|  |  |  |  |  |  |  |  |  |
| Serranidae | *Cephalopholis fulva* | MCAR | 218 | No | 0 | 0.23 ± 0.07 | 0 | 0.06 ± 0.02 |
|  | *Diplectrum* sp. | MCAR | 55 | No | 0 | 0.11 ± 0.05 | 0 | 0.01 ± 0.01 |
|  | *Alphestes afer* | MCAR | 30 | No | 0.02 ± 0.02 | 0.02 ± 0.02 | 0.01 ± 0.01 | 0.01 ± 0.01 |
|  | *Epinephelus marginatus* | MCAR | 300 | No | 0.02 ± 0.02 | 0 | 0.01 ± 0.01 | 0 |
|  | *Epinephelus morio* | MCAR | 300 | No | 0.15 ± 0.05 | 0.01 ± 0.01 | 0.12 ± 0.05 | 0.04 ± 0.04 |
|  | *Mycteroperca acutirostris* | MCAR | 110 | No | 0.02 ± 0.02 | 0 | 0.01 ± 0.01 | 0 |
|  | *Mycteroperca bonaci* | MCAR | 70 | No | 0.83 ± 0.13 | 0.02 ± 0.02 | 2.14 ± 0.56 | 0.02 ± 0.02 |
|  | *Rypticus saponaceus* | MINV | 68 | No | 0.02 ± 0.02 | 0 | 0.01 ± 0.01 | 0 |
|  | *Serranus atrobranchus* | MINV | 219 | No | 0.02 ± 0.02 | 0 | 0.01 ± 0.01 | 0 |
|  | *Serranus baldwini* | MINV | 80 | No | 0 | 0.06 ± 0.02 | 0 | 0.01 ± 0.01 |
|  | *Serranus chionaraia* | MINV | 90 | No | 0 | 0.13 ± 0.06 | 0 | 0.01 ± 0.01 |
|  | *Serranus phoebe* | MINV | 400 | No | 0 | 0.18 ± 0.08 | 0 | 0.01 ± 0.01 |
|  | *Serranus* sp.1 |  |  |  | 0 | 0.21 ± 0.05 | 0 | 0.01 ± 0.01 |
|  | *Serranus annularis* | MINV | 70 | No | 0 | 0.07 ± 0.03 | 0 | 0.01 ± 0.01 |
|  |  |  |  |  |  |  |  |  |
| Chaetodontidae | *Chaetodon sedentarius* | SINV | 92 | No | 0 | 0.10 ± 0.04 | 0 | 0.01 ± 0.01 |
|  | *Chaetodon striatus* | SINV | 65 | No | 0.42 ± 0.09 | 0.08 ± 0.05 | 0.01 ± 0 | 0.01 ± 0.01 |
|  | *Prognathodes brasiliensis* | SINV | 65 | Yes | 0 | 0.02 ± 0.02 | 0 | 0.01 ± 0.01 |
|  |  |  |  |  |  |  |  |  |
| Pomacanthidae | *Centropyge aurantonotus* | HERB | 200 | Yes | 0 | 0.01 ± 0.01 | 0 | 0.01 ± 0.01 |
|  | *Holacanthus ciliaris* | SINV | 120 | No | 0.02 ± 0.02 | 0.09 ± 0.03 | 0.01 ± 0.01 | 0.02 ± 0.01 |
|  | *Holacanthus tricolor* | SINV | 92 | No | 0 | 0.07 ± 0.03 | 0 | 0.01 ± 0 |
|  | *Pomacanthus arcuatus* | SINV | 30 | No | 0.32 ± 0.07 | 0.10 ± 0.04 | 0.14 ± 0.04 | 0.13 ± 0.06 |
|  | *Pomacanthus paru* | SINV | 100 | No | 1.13 ± 0.27 | 0.30 ± 0.08 | 1.21 ± 0.34 | 0.3 ± 0.09 |
|  |  |  |  |  |  |  |  |  |
| Malacanthidae | *Malacanthus plumieri* | MCAR | 153 | No | 0 | 0.37 ± 0.10 | 0 | 0.14 ± 0.04 |
|  |  |  |  |  |  |  |  |  |
| Haemulidae | *Anisotremus surinamensis* | MINV | 60 | No | 0.03 ± 0.02 | 0 | 0.07 ± 0.06 | 0 |
|  | *Anisotremus virginicus* | MINV | 40 | No | 0.70 ± 0.24 | 0.09 ± 0.04 | 0.11 ± 0.05 | 0.04 ± 0.02 |
|  | *Haemulon album* | MINV | 60 | No | 0 | 0.01 ± 0.01 | 0 | 0.01 ± 0.01 |
|  | *Haemulon aurolineatum* | MINV | 70 | No | 9.33 ± 2.64 | 1.98 ± 0.89 | 0.24 ± 0.07 | 0.09 ± 0.04 |
|  | *Haemulon parra* | MINV | 60 | No | 0.02 ± 0.02 | 0 | 0.01 ± 0.01 | 0 |
|  | *Haemulon plumieri* | MINV | 70 | No | 1.23 ± 0.36 | 0.47 ± 0.09 | 0.29 ± 0.11 | 0.16 ± 0.03 |
|  | *Haemulon squamipinna* | MINV | 40 | Yes | 0.03 ± 0.03 | 0.31 ± 0.31 | 0.01 ± 0.01 | 0.01 ± 0.01 |
|  |  |  |  |  |  |  |  |  |
| Lutjanidae | *Lutjanus analis* | MCAR | 95 | No | 0.03 ± 0.02 | 0 | 0.03 ± 0.02 | 0 |
|  | *Lutjanus jocu* | MCAR | 70 | No | 0.25 ± 0.08 | 0.01 ± 0.01 | 0.29 ± 0.11 | 0.02 ± 0.02 |
|  | *Lutjanus synagris* | MCAR | 400 | No | 0.08 ± 0.08 | 0.03 ± 0.02 | 0.01 ± 0.01 | 0.02 ± 0.02 |
|  | *Ocyurus chrysurus* | MCAR | 180 | No | 14.62 ± 1.56 | 1.42 ± 0.23 | 1.7 ± 0.2 | 0.38 ± 0.07 |
|  | *Rhomboplites aurorubens* | MCAR | 300 | No | 0 | 0.87 ± 0.49 | 0 | 0.23 ± 0.12 |
|  |  |  |  |  |  |  |  |  |
| Ephippidae | *Chaetodipterus faber* | MINV | 35 | No | 0.02 ± 0.02 | 0 | 0.02 ± 0.02 | 0 |
|  |  |  |  |  |  |  |  |  |
| Acanthuridae | *Acanthurus bahianus* | HERB | 71 | Yes | 1.88 ± 0.46 | 0.16 ± 0.06 | 0.23 ± 0.08 | 0.02 ± 0.01 |
|  | *Acanthurus chirurgus* | HERB | 70 | No | 0.25 ± 0.20 | 0.07 ± 0.04 | 0.07 ± 0.06 | 0.02 ± 0.01 |
|  | *Acanthurus coeruleus* | HERB | 71 | No | 1.28 ± 0.52 | 0.11 ± 0.05 | 0.26 ± 0.13 | 0.06 ± 0.04 |
|  |  |  |  |  |  |  |  |  |
| Sparidae | *Calamus* sp. | MINV | 86 | No | 0.15 ± 0.06 | 0.49 ± 0.07 | 0.07 ± 0.03 | 0.21 ± 0.03 |
|  |  |  |  |  |  |  |  |  |
| Balistidae | *Balistes capriscus* | MINV | 100 | No | 0 | 0.02 ± 0.02 | 0 | 0.01 ± 0.01 |
|  | *Balistes vetula* | MINV | 111 | No | 0.10 ± 0.04 | 1.68 ± 0.23 | 0.16 ± 0.07 | 1.82 ± 0.24 |
|  |  |  |  |  |  |  |  |  |
| Monacanthidae | *Aluterus monoceros* | OMNI | 72 | No | 0 | 0.16 ± 0.10 | 0 | 0.09 ± 0.06 |
|  | *Aluterus scriptus* | OMNI | 120 | No | 0 | 0.02 ± 0.02 | 0 | 0.03 ± 0.02 |
|  | *Cantherhines macrocerus* | OMNI | 62 | No | 0.07 ± 0.04 | 0.24 ± 0.06 | 0.04 ± 0.03 | 0.12 ± 0.03 |
|  | *Monacanthus ciliatus* | OMNI | 50 | No | 0 | 0.01 ± 0.01 | 0 | 0.01 ± 0.01 |
|  |  |  |  |  |  |  |  |  |
| Tetraodontidae | *Canthigaster figueiredoi* | OMNI | 66 | Yes | 0.03 ± 0.02 | 0.03 ± 0.02 | 0.01 ± 0.01 | 0.01 ± 0.01 |
|  | *Lagocephalus laevigatus* | MCAR | 180 | No | 0 | 0.26 ± 0.08 | 0 | 0.16 ± 0.05 |
